# The emerging roles of eosinophils in immune regulation in health and disease

**DOI:** 10.3389/fimmu.2026.1652583

**Published:** 2026-07-09

**Authors:** William Zhang, Marie Caligaris, Surabhi Tiwary, Dan Nicolau

**Affiliations:** King’s College London, London, United Kingdom

**Keywords:** eosinophils, immune regulation, inflammatory response, Th2 (type-2) immune responses, tissue remodeling and repair

## Abstract

Eosinophils are evolutionarily conserved granular innate cells that can trace back to 100 million years to the emergence of gnathostomes, which now is involved in regulation of diverse homeostatic conditions and diseases in mammals. They have traditionally been associated with Th2 responses, primarily in eosinophilic allergic asthma and helminth parasitic infections. These Th2 responses are correlated with basic cationic proteins and specific cytokines release from preformed eosinophilic vesicles, associated with tissue damage and remodeling. Consequently, eosinophils have historically been characterized as potent cytotoxic effector cells. In human patients, their high blood count has been linked to clinical concerns and necessary further patient evaluations. However, this reflects only a cursory understanding of eosinophils, as their involvement in a broad and diverse range of disorders has recently emerged. Indeed, eosinophils extend beyond traditionally ascribed Th2 responses, which appear to be both global and pivotal in immune regulation contexts. Its role varies from speculative controlled embryological tissue modeling, to tissue regeneration post-injury, uncontrolled neoplastic development, novel Th2 eosinophilic gastrointestinal disorders, reproductive homeostasis and SARS-CoV-2 in a non-exhaustive list of its emerging association with health and disease. This review evaluates the emerging roles of eosinophils which are both pro- and anti-inflammatory, dependent on disease context and microenvironment. Our knowledge of the novel and diverse roles of eosinophils has been applied to the emergence of targeted, and successful clinical therapies to a range of diseases, opening doors to extend assessment on their potential roles in the original embryological modeling and development. Collectively, diverse modern evidence indicates that eosinophils—an ancient evolutionarily conserved cell lineage—has an imperative function in immune networks.

## 1 Introduction

Eosinophils are a subtype of terminally differentiated (1) bilobed innate granular leukocytes (2), present in all vertebrates (3, 4) and typically constitutes 1-5% of all circulating human white blood cells (1, 5, 6). First described in 1870s by Paul Ehrlich (1, 5): it is estimated that the eosinophil cell lineage dates back hundred million years (7) to the origin of jaws, making them older than the adaptive immune system.

Eosinophils’ role in immune responses have long been attributed to traditional T-helper type 2 (Th2) functions, primarily in allergic respiratory diseases, to which asthma is a major contributor, and in host defense, especially against parasites such as helminths. Conversely, eosinophils have historically been characterized as potent cytotoxic effector cells, through the release of preformed cationic protein-filled vesicles leading to epithelial cell damage and tissue remodeling in inflammatory settings. However, the list of diseases in which eosinophil involvement has been attributed to has grown to include a broad and diverse range of disorders, in which eosinophils function as both pro- and anti-inflammatory innate cells. Among others, eosinophils are highly involved in the development of cancer, during which they can act as both anti-tumorigenic mediators, through tumor-cell recognition and adhesion, enhancing cytotoxicity and decreasing metastatic spread, as well as pro-tumorigenic contributors through the increase in tumor angiogenesis and metastasis, as well as enhancing mechanisms of immune evasion. These dual roles are cancer- and stage-specific and hugely dependent on their tumor microenvironment. In addition, eosinophil functions have extended to include roles as key facilitators of controlled tissue repair and regeneration following injury and speculatively in embryological development, in eosinophil gastrointestinal inflammatory disorders, reproductive homeostasis, obesity and type-2 diabetes and SARS-CoV-2 among others in a non-exhaustive list of eosinophil-mediated diseases.

This critical literature review will first briefly summarize the traditionally ascribed roles of eosinophils in respiratory allergic inflammation and parasitic defense. It will then go onto discuss the emerging functions of eosinophils in both health and diseases, focusing on controlled injured tissue regeneration, evolving pro- and anti-tumorigenic roles in cancer and its prognosis, as well as in eosinophil esophagitis, a novel and developing Th2-mediated illness with rapidly rising prevalence worldwide.

## 2 Origin, evolution and biology of eosinophils

To initiate discussion of eosinophils’ roles in immunity, its evolutionary origin and biology should be dissected first. Eosinophils develop in the bone marrow from hematopoietic stem cells (8, 9) under the regulation of specific cytokines, primarily interleukin-5 (IL-5), which appears to be the most crucial and eosinophil-specific (10) mediator of growth, differentiation and proliferation, as well as inerleukin-3(IL-3), Granulocyte-macrophage colony-stimulating factor (GM-CSF) and the GATA-1 transcription factor (5, 9, 11). Once committed to the eosinophil lineage, CD34+/IL-5Rα+ (interleukin-5 receptor alpha) eosinophils (12) are released into the peripheral blood, where they circulate under homeostatic conditions (1) and migrate to specific tissues (primarily the gastrointestinal tract, lungs, uterus and adipose tissue (1)) following chemotaxis gradients of chemokines including eosinophil-specific C-C motif chemokine ligand 11 and 24 (CCL11/24) (previously called eotaxin-1 and eotaxin -2) and cytokines, particularly IL-5, during inflammatory conditions (1, 11, 13).

Eosinophils express a wide variety of receptors on their cell surface, including complement receptors and pattern recognition receptors (PRRs) - allowing them to act as sentinels (5) and sense pathogens (10) – as well as adaptive immune interaction Fc receptors (14, 15) and eosinophil-specific CD34, IL-5Rα (8, 10, 15) and the CC-chemokine receptor-3 (CCR3) (15) receptors. Like other granulocytes, eosinophils possess cytoplasmic vesicles containing four major groups of cationic proteins: major basic protein 1 (MBP-1), eosinophil peroxidase (EPX), eosinophilic cationic protein (ECP), and eosinophil-derived neurotoxins (EDN) (3, 5), as well as a number of cytokines, such as interferon-gamma (IFN-γ), tumor necrosis factor (TNF), interleukin-4(IL-4) and interleukin-6 (IL-6) (3, 16). Human eosinophil granules also contain galectin-10 (14, 15, 17), a cytosolic, non-cationic hydrophobic crystal protein (14, 17) that represents 7-10% of eosinophil cellular protein contents (14, 15), and plays a role in the formation of eosinophil extracellular traps (EETs). Following pathogen recognition in tissues or in the blood, eosinophils release their preformed granules (15) as a defense mechanism against the pathogen. Studies by Doyle and colleagues (18) have shown that the granule proteins are not only key features of granulocytes but, in the case of eosinophils, they are necessary for eosinophilopoeisis. In double MBP-1-/-/EPX-/- knockout mice, peripheral blood eosinophils are almost absent, whereas other granulocytes, such as basophils, appear unaffected (18, 19). MBP-1 and EXP gene expression are rheostats of the eosinophil lineage, with their absence resulting in the loss of eosinophil-committed progenitors (3, 18, 20). Upon differentiation, eosinophils can be categorized into two groups based on their phenotypic activities and roles in immune responses (14). While a definitive consensus is still unfolding, current literature proposes a conceptual framework classifying eosinophils into distinct E1 and E2 subpopulations based on their polarization towardTh1-type or Th2 type immune networks, respectively (14).

## 3 Traditionally known roles of eosinophils Th2 immunity

**Figure 1:**
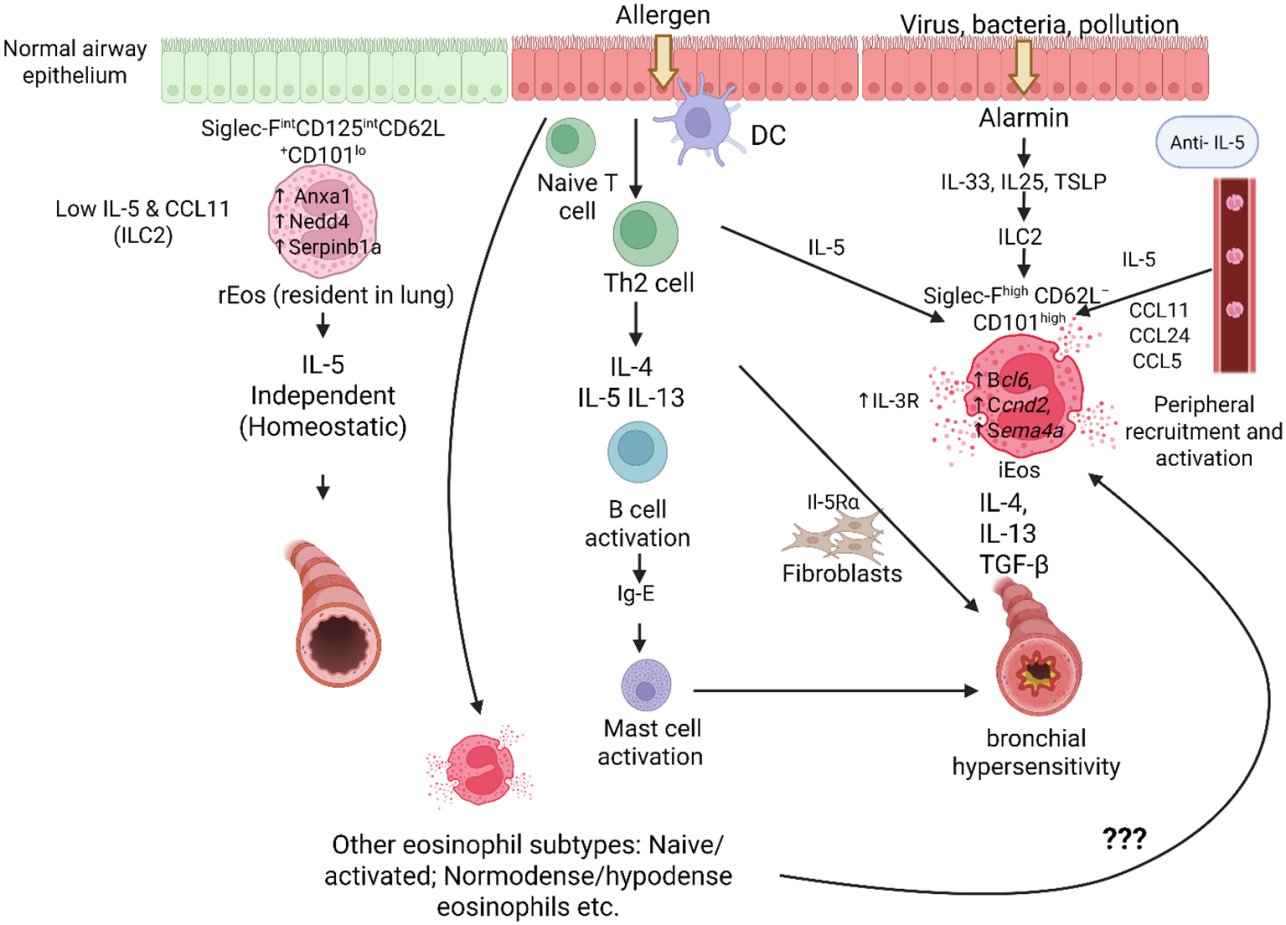
Schematic representation of eosinophil subpopulations and activation pathways. Resident Eosinophils(rEos) are homeostatic, steady-state cells that function independently of interleukin-5 (IL-5) within lung tissues to support tissue repair and baseline immunoregulation. In contrast inflammatory(iEos) are recruited and activated by a cascade of upstream cytokines and chemokines, upregulating the expression of B-cell lymphoma 6, cyclin D2 (Ccnd2), and semaphoring 4(Sema4). The activation drives the production of interleukin-4(IL-4), interleukin-13(IL-13), and transforming growth factor-beta (TGF-β), ultimately promoting bronchial hypersensitivity and fibroblast-mediated tissue remodeling. In parallel, allergens provoke hypersensitivity via a classic type 2 T helper (Th2) cell-mediated immune response; this pathway produces IL-4, IL-5, and IL-13, stimulating B cells to synthesize immunoglobulin E (IgE) and activating mast cells. Whether the distinct eosinophil phenotypes generated across these discrete activation pathways share overlapping functional features or represent identical cellular lineages remains understudied, as indicated by the question mark denoting key areas for future investigation (38, 43).

The classified eosinophil types have distinct roles in health and disease (as shown in **Figure 1**), but they have extensively and traditionally been attributed to their pathogenesis in Th2 responses, principally their importance in atopic diseases such as allergies and asthma (6, 8, 21), as well as in the clearance of helminths and other parasitic, bacterial and viral infections (6, 14, 21). Th2 responses are characterized by IL-4 production, and consequently, cells capable of spontaneously producing and secreting IL-4, such as eosinophils, mast cells and basophils are recruited to and activated at the inflammatory site, especially in the theme of allergic respiratory inflammation.

### 3.1 Allergic respiratory inflammation

Allergic respiratory inflammation, particularly asthma, is one of the most common chronic respiratory diseases worldwide and is driven by Th2 type responses described above (22). Based on the presence of eosinophilic infiltration, asthma can be broadly classified into two major phenotypes: type-2 high (eosinophilic) and type-2 low (non-eosinophilic). Within the broad type-2 high spectrum, there are distinct underlying subsets: atopic asthma, which is an allergic response mediated by adaptive Th2 cell differentiation, and non-atopic eosinophilic asthma, which is non-allergic and driven primarily by innate lymphoid cells (ILC2s) (23). Healthy patients hold an accumulation rate of eosinophils to the lungs of approximately 30 eosinophils/min/mL, whereas accumulation rates of type-2 high asthmatics to the lungs are on average 10–100 fold greater (15).

In response to stimuli, such as allergic respiratory inflammation, eosinophils accumulate in the lungs, via chemogradient of CCL11, CCL24 and CCL5 (previously called RANTES) (24) and function as pivotal modulators in Th2 responses (21), and in the activation of T-helper lymphocytes (21, 25). Jacobsen et al. (26) showed that eosinophils are capable of rapid polarization and activate Th2 lymphocytes (26, 27) leading to an increased production of Th2 cytokines (28), primarily IL-5. In return, this induces the production and accumulation of eosinophils (eosinophilia) to the inflammatory site (23, 26, 29), as well as IL-4 and IL-13, which trigger increased production of Immunoglobin E(IgE) and Immunoglobin G1(IgG1) (26) through B cell antibody class switching, and are responsible for bronchiole hypersensitiveness (26). Furthermore, eosinophils have the capacity to act as antigen presenting cells (30), through interacting with the adaptive system and activating naïve CD4+ T cells (30–32). 

Given such complications of eosinophils, studies later found that the release of their preformed granules in the lungs is associated with airway remodeling (33, 34) and pulmonary dysfunction (35), the symptoms of eosinophilic asthma. Using allergenic-naïve double-transgenic mice expressing both T-cell derived IL-5 and lung epithelial CCL24, severe asthma was observed (34). This was also pathogenically represented by mucus hypersecretion, epithelial desquamation with remodeling and smooth muscle hyperplasia, as well as large volumes of eosinophil degranulation (34). Such remodeling is produced by eosinophilic granule release containing proteases, further detailed below.

**Figure 2:**
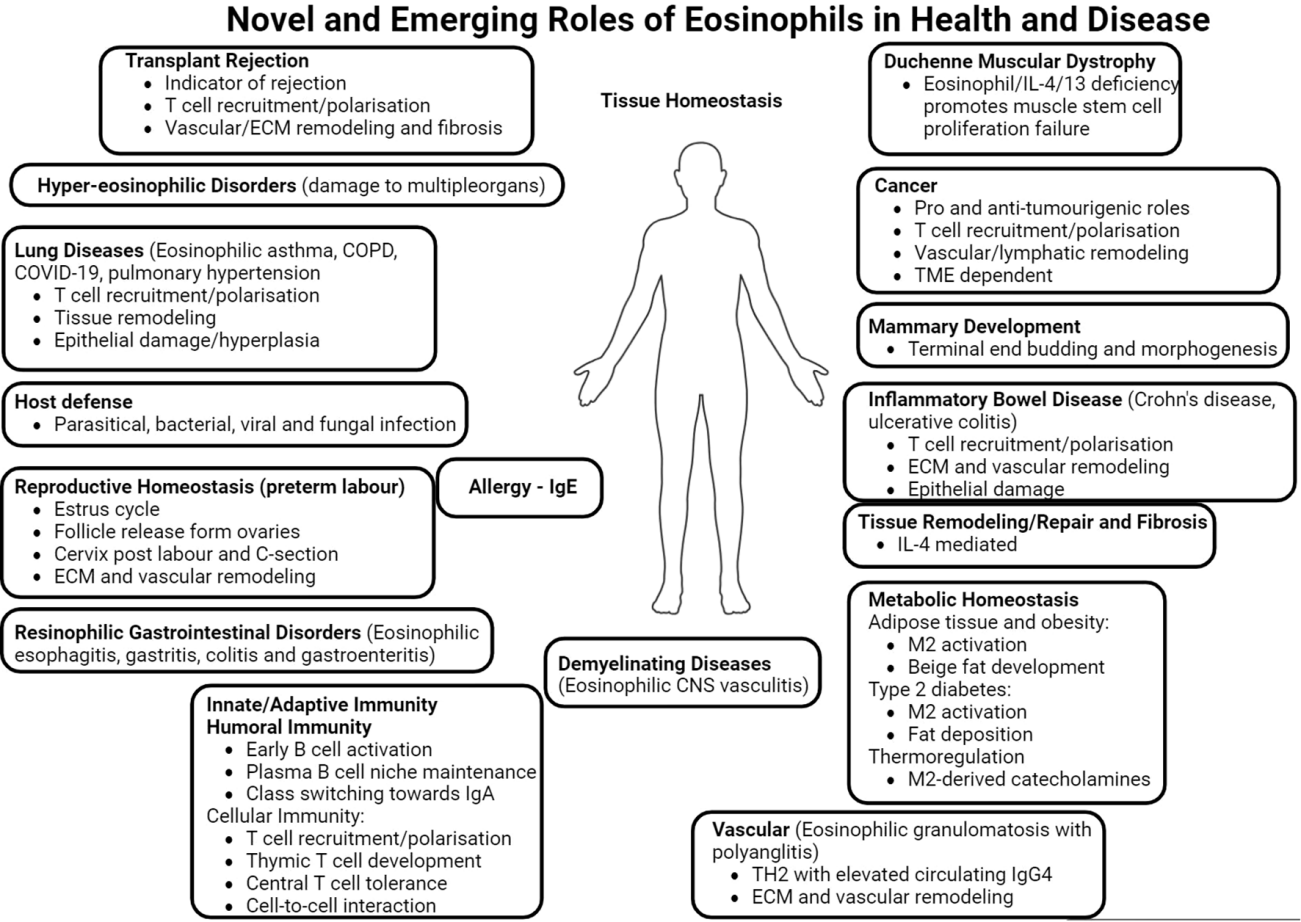
A schematic representation of the vast array of novel and broad functions of eosinophils in health and disease.

Traditional models propose that the release of MBP-1 and EPX granular proteins damage the lung epithelial cells by affecting the lipid bilayer membrane (36), while MBP-1 causes bronchoconstriction (37, 38) and contributes to the release of histamine from activated mast cells (39). Additionally, once released from their vesicles, galectin-10 proteins crystallize to form Charcot-Leyden (38, 40, 41), creating an eosinophil extracellular trap cell death as a result of bronchial eosinophil accumulation (40). Lastly, Halwani et al. (24) and Minshall et al. (42) highlighted eosinophils as the fundamental producers of TGF-β in airways (24), specifically during an allergic asthmatic immune response, where the growth factor contributes to airway remodeling (24, 38, 42) and reticular lamina thickening in lungs (42), also schematically represented in **Figure 2** (38).

Eosinophil induces cytotoxicity primarily by releasing their cationic and toxic granule contents at inflammation sites (44), despite their blood count prompting clinical concern (14, 20). Price et al. (45) highlighted the correlation between increased peripheral blood eosinophil count and the disease severity (45). Also pathologically observed by increased epithelial damage, airway remodeling, obstruction and bronchiole hypersensitivity (14, 20). Beside eosinophilic granule release, eosinophil-derived exosomes, Eosinophil Extracelluar Traps (EETs), and cytokine signaling cascades are secondary processes facilitating tissue remodeling. Specifically, EETs have recently been found to be correlated with cancer prognosis as it’s involved in tumor microenvironment remodeling (20, 46).

Pertaining on further elucidation of eosinophils’ identities, studies of wildtype (C57BL/6 and BALB/c) and inflamed A_dblGATA_ mice models have revealed two phenotypically different subcategories of eosinophils (47), with different functions in asthmatic inflammatory response. Hence, the recent concept of: Lung resident eosinophils (rEos) and inducible peribronchial eosinophils (iEos) arose, representing their homeostatic and pro-inflammatory nature respectively. The former is Siglec-F^int^ CD62L + CD101^low^, IL-5-independent, which are located in parenchymal cells and implicated in negative inflammatory regulation in the lungs (43); while the later migrates to inflammatory sites, which are Siglec-F^high^ CD62L-CD101^high^, IL-5-dependent and express pro-inflammatory genes (43). A key signaling pathway underlying the transition to iEos was recently demonstrated by Zhu et al.,twho showed that TGF-β, directly induces CD101 expression on eosinophils in an experimental model of eosinophilic asthma (48). However, two other distinct mechanisms exist, first of which is triggered by release of alarmins, resulting from environmental stimulus such as pollution, allergens. These alarmins stimulates IL-25, IL-33 and TSLP release as demonstrated in **Figure 2** (49), which alongside Eicosanoids activate ILC2 that respond to alarmin and leukotrienes. Consequently, downstream cytokines: IL-4, IL-5 and IL-13 which are responsible for airway eosinophilia and tissue damage, inflammation are produced. Distinct from that, the second mechanism is facilitated by IL-4 that differentiates naïve CD4+ T cells into Th2 cells, in turn promoting IL-5 and 13 secretion. This triggers a cascade of signaling molecules including PI3K, JAK2, STAT1,3 and 5, NF-kB and MAPK, alongside inducible nitric oxide synthase expression, FENO production, leading to bronchial hyperactivity and muscle cell contraction (50). These mechanisms outline the activation of inducible ‘pro-inflammatory’ eosinophils.

Conversely, there are homeostatic eosinophils (resident eosinophils) which are immune regulators of local immunity/remodeling and repair in health and diseases given their localized affection. In the respiratory tract, small proportion of eosinophils reside in healthy lungs. These eosinophils express Anxa1, Nedd4, Runx3, Serpinb1a, Ldlr and Metrnl transcripts, which contribute to immune regulation and tissue homeostasis (51). Their localization in the local airway epithelial cells is achieved by constitutively expressing CCL11 at low levels, keeping homeostatic eosinophils in its niche. While its maintenance is achieved through basal level IL5 production by ILC2, which do not trigger eosinophil activation, but rather provide a steady low-profile signal. Although various eosinophil subtypes have been recognized, the field still lacks clarification on their transitional identity, questions remain as whether these groups represent distinct eosinophil subtypes, or rather a continuum of activation state transitions. It’s evident that eosinophils are tissue microenvironment dependent, but further work is required to determine inter-connections between these subtypes. Once they have been elucidated, subtype markers could aid clinical profiling of patients to allow patient-specific treatments, also potentially revealing unique anomalies.

However, results of IL-5 dependent differentiation in subcategories of human eosinophils requires further research given its clinical translational value for targeted treatment. Overall, murine models currently dominate the field as human eosinophil subpopulations although growing, remain poorly defined under steady-state conditions. Recent study by Albinsson et al. identified transcriptionally and phenotypically distinct eosinophil subtypes in infants. Where thymic eosinophils demonstrated increased CD31, CD38 and CD183 expression, also promoting CD4+ single-positive thymocyte development, and suppressing CD8+ single-positive thymocyte development. Such finding suggests a role in influencing T cell lineage commitment. Therefore, relevant studies are emerging with many suggesting similar plasticity programs in murine models and *in vivo* (52). Recent advances in spatial transcriptomics technology could aid eosinophils phenotype understanding in their microenvironmental tissue context. Such application have only recently been adopted for profiling eosinophils under various contexts such as in COPD, which successfully visualized eosinophil distribution to aid spatial location examination with histological features (53); while other studies identified active and basal eosinophils subtypes in colitis patients, finding that IL-33 and IFN-γ induce active eosinophil accumulation in inflamed colon (54) using spatial transcriptomics tools. Such adoption is also desirable in the context of eosinophilic asthma, as it will provide single-cell and inter-cell pathways to further dissect eosinophil communication within asthmatic micro-environment.

Despite recent asthma treatments evolving towards targeting the central IL-5 eosinophil pathway which is inter-connected with iEos and rEos (55), its efficacy remains unstable as it depletes both subtypes. These include anti-IL-5 antibody treatments:mepolizumab and reslizumab, and the IL-5Rα-directed cytosolic monoclonal antibody medication benralizumab, which acts to deplete circulating and resident eosinophils numbers through antibody-mediated cytotoxicity (55). Leckie et al. showed that IL-5-blocking monoclonal antibody proved an efficient treatment through reducing airway tissue and bone-marrow eosinophils by 55% and 52% respectively over a 20-week treatment course (56). Benralizumab treatment, mainly used in severely asthmatic patients showing eosinophil-induced inflammation, was trialed in 2020 in two key drug trials, SIROCCO (AstraZeneca and Kyowa Hakko Kirin funded) and CALIMA (AstraZeneca funded) and showed substantial patient benefit with no increased risk of associated infection. Thereafter, GALATHEA and TERRANOVA trials (both AstraZeneca and MedImmune LLC funded) for COPD patients (55) both trialed Benralizumab, and similar results to the eosinophilic asthma trials were yielded. However, as will be discussed further in this review, eosinophils also have critical and beneficiary roles in host protection as well as tumor cell killing. As a result, the effects of eosinophil depletion as treatment for severe respiratory conditions need to be further evaluated in a risk/benefit manner in human patientsas over-depletion could be a risk disrupting homeostasis (55). Hence, targeted therapy of specific eosinophil subtype through targeting other markers, or pathways could be beneficial, although requires proof of concept. It’s crucial to acknowledge the differences in mice model and in humans as they fail to be translated in clinic. Key differences include amplified involvement of Leukotriene B4(LTB4) and specialized pro-resolving mediator in mice compared to Leukotriene C4(LTC4) in humans, while impact of endocannabinoid 2-arachidonoyl-glycerol(2-AG) has diminished effects compared to human. Additionally pro-resolving mediators are more efficiently produced in mice, alongside prostaglandins which are more significantly produced in mice. Collectively, mice model present more neutrophil chemoattractant (high LTB4), resolution of inflammatory response (100 fold less prominent production of 2-AG), and hence they show different pathways, where humans use 2-AG and LTC4, while mic us LTB4 and prostaglandin, a key underlying biological difference to consider for translational studies.

### 3.2 Parasitic infection

Allergic responses are not the only field that eosinophils plays a pivotal role in, striking similarities are also shared with specific helminth infections, which characteristically drive a Th2 response (19). However, this immune profile is highly paraite-specific, as eosinophil induction and functionality vary significantly depending on the invading species and its life-cycle stage (15), although the precise agenda of these innate granulocytes in immune defense varies across different worm species. In the context of macroparasites like helminths, eosinophils can directly mediate larval or adult worm damage through cationic degranulationInterestingly, during a parasitic infection caused by *Nippostrongylus brasiliensis* helminth, seminal studies by Taliaferro and Sarles in the 1930s (57) demonstrated that eosinophils robustly accumulate at the local site of infection (58). In this context, eosinophils can directly mediate the killing of parasites through cationic degranulation (57, 59) of MBP-1 (60) and reactive oxygen species (ROS) (61)as well as through antibody-dependent pathways (19, 62, 63).

Studies using IL-5^-/-^ and ΔdblGATA mice, which contain a deletion of the GATA site of the GATA1 promoter and therefore eosinophil cell line depletion (19, 64) and lead to eosinophilopoeisis-deficient animals, showed an increased number of *Nippostrongylus brasiliensis* helminths in infected lungs, as compared with wild type mice (19, 64). Findings by Knott et al. (64) confirmed that eosinophil-deficient mice have an impaired resistance to the *N. brasiliensis* helminths, and that therefore, in this setting, eosinophils have an anti-parasitic role.

Specific research also studied filarial infection (Wuchereria bancrofti, Brugia malayi, Loa loa, and Onchocerca volvulus), a subset of helminth conditions eliciting eosinophilia as its hallmark (65). Early studies by Hall et al. adopted mechanistical mouse models to provide direct proof of filaria-induced lung pathology mechanism. The study first sensitized C57BL/6 mice with killed B.malayi microfilariae, and challenged them with the live worm, and the mice mirrored the human disease perfectly. Through observation against a comparative group of mechanistic knockout IL-5 deficient mice, which developed no eosinophilia, direct proved that filaria-induced lung pathology is caused by IL-5 dependent eosinophil-mediated immunopathological event, rather than the worm itself. Extending from this, landmark studies by Nutman demonstrated innate lymphoid cells (ILCs) expanded in both mouse models and human filarial infections (66–68), inducing significant amount of IL-5 and 13 preceding classical Th2 response onset, alongside eosinophils populations expansions (69). This is later reinforced by findings of thymic stromal lymphopoietin, IL-25, and IL-33, which initiate response against tissue damage caused by the infection. In response, IL-4, 9, 10 and 13 are produced to support expansion of ILC type 2, eosinophils, alternatively activated macrophages and Th2 cells, initiating the eosinophilic effector response. Such response involves the release of crystalloid, small, primary granules and secretory vesicles, containing key proteins such as Charcot-Leyden crystal (CLC) forming proteins, and eosinophil peroxidase which aids parasite killing by reactive oxygen species productions (70). However, there are differential presence of CLC forming proteins, which are found in human but not murine eosinophils. Furthermore, tropical pulmonary eosinophilia has been recorded to develop in human patients infected with W.bancrofti and B.malayi, represented by elevated levels of 3000 eosinophils/ul (71, 72). In conjunction, despite commonalities in eosinophilia responses in general, deviations between mechanisms in human and mice are present, demanding further validation of translational studies. This is indeed true as Legrand et al. conducted a randomized, double-blind, placebo-controlled clinical trial, where depletion of eosinophil counts with reslizumab (anti-IL-5) prior to anti-parasitic treatment (Diethylcarbamazine citrate) did not alter microfilarial clearance. The study is a pilot proof-of-concept study that suggest such clearance can occur without the presence of high absolute eosinophil count, deviating the traditional *in vitro* “killing” dogma from *in vivo* human realities (73). Interestingly, environmental factors were studied in comparative groups of temporary residents who contracted Loa Loa and indigenous individuals (END) to endemic area. Differing responses were developed by travelers (TR), showing hyper-reactive Th2 response with severe Calabar swelling and massive blood eosinophilia (130138 vs 72388 ng/ml eosinophil granule proteins; 3.9 vs 1.9 ng/ml eosinophil peroxidate in TR and END respectively), despite low Loa Loa counts. Whereas indigenous individuals were microfilariae positive but demonstrated suppressed eosinophil symptoms and less complications (74). Therefore, eosinophil activation and degranulation state are found to significantly differ between groups, although for unknown reasons, it opened an exciting path to study regulatory mechanisms responsible for the differences.

However, more recently, the actions of eosinophils during chronic nematode infections have been called into question, suggesting that eosinophils and certain tissue-dwelling helminthsmay have a more symbiotic relationship than previously thought, with eosinophils supporting larval persistence (75, 76). Fabre et al. (75) used transgenic PHIL mice, where a sequence of diphtheria toxic A chain is added into the eosinophil peroxidase (EPX) locus, and ΔdblGATA mice, both deficient in eosinophils, to explore this hypothesis. They found that the presence of eosinophils in muscle limited the killing of *Trichinella spiralis* and promoted the formation of muscle nurse cells necessary for T.spiralis larval survival. In contrast, a decrease in the number of muscle nurse cells in infected hosts (75) reflected the absence of eosinophils. In addition, eosinophil ablation resulted in the recruitment of Th1/M1 type macrophages, which express inducible nitric oxide synthase and promote the killing of the parasite encysted larvae as well as muscle cell necrosis. a decrease in the number of muscle nurse cells in infected hosts.

On the other hand, gastrointestinal tract infection with T. spiralis helminths showed no alteration in infection of PHIL and ΔdblGATA mice compared to wild type (75). It is therefore possible that although eosinophils are capable of both pro-inflammatory and helminth symbiotic mediation, this role is highly divergent depending on the invading worm species, the tissue or organoid infected, as well as the nematode’s life-cycle stage (77), in other words, on the distinct infection context.This is an active area of research and further work is both needed and ongoing.

## 4 The emerging roles of eosinophils in health and disease

Although the roles of eosinophils in regulation in health and disease have long been associated with their destructive nature in Th2 responses (20), more recent research has provided evidence for their much wider functions in disease regulation and homeostasis. As schematically mapped out in **Figure 2**, the expanding landscape of eosinophil biology can be broadly bifurcated into two functional domains: their role as destructive drivers of explicit clinical pathologies (diseases) and their functions as homeostatic regulators during physiological conditions or localized immune complications. Parallel to traditional paradigms which focuses on eosinophil’s cytotoxic contributions, modern body of evidence demonstrates their necessity in non-disease physiological conditions. These include coordination of metabolic homeostasis, innate/adaptive immune network signaling, tissue repair and host-graft immune conflict which define acute organ transplant rejection. This section reviews these dual paradigms, viewing controlled physiological modeling versus uncontrolled disease states.

### 4.1 Physiological conditions: controlled tissue modeling and repair

Emerging research has established that the beneficial roles of eosinophils in controlled tissue repair and modeling are mechanistically driven by an IL-4 regenerative response, as demonstrated in murine models of partial hepatectomy (78). The local expression of CCL5 in the inflammatory foci acts as a chemoattractant, recruiting eosinophils, which are the primary source of IL-4 expressing cells in injured tissue. IL-4 functions to activate resident fibrocyte-adipocyte progenitors (FAPs), which promote the proliferation and regeneration of damaged tissue cells (79). Additionally, the expression of Th2 cytokines at the inflammatory site insures myogenesis from FAPs, through inhibition of their differentiation into adipocytes (79). In addition, expression of vascular endothelial growth factor (VEGF), a pro-angiogenic mediator, by eosinophils promotes the formation of new blood vessels needed in tissue repair. These roles have been studied in regeneration of the liver through proliferation of hepatocytes, as well as in muscle, such as following myocardial infarction, where production of IL-4 and mEar1 (murine ortholog of human ECP) confer protection through limiting cardiomyocyte apoptosis and increasing their proliferation, regulating fibroblast action, modulating inflammatory cell infiltration and adhesion and ensuring healing of the infarct tissue (9). During physiological conditions, TGF-β and expression of its receptor is essential for wound healing, tissue and immune homeostasis, and its absence is commonly found in chronic wounds in patients with wound chronicity and unhealing. All isoforms of TGF-β increase dramatically after wounding, promoting re-epithelialization, tissue angiogenesis and fibroblast activation. Apart from skin, it’s also induced in non-parenchymal cells during mice liver regeneration, which interestingly exhibit upregulation of all TβR types, enhancing responsiveness to TGF-β and prevent uncontrolled cell proliferation. However, numerous cells including eosinophils, neutrophils and macrophages in inflammatory conditions can contribute to excessive TGF-β production in asthmatic patients, contributing to airway remodeling. Although contradictory functions of TGF-β was revealed that can either enhance or suppress eosinophil activities. For instance, in dermatitis-like skin lesions, TGF-β1 was demonstrated in NC/Nga mice to suppress IFN- γ, also suppressing atopic skin lesions (80), promoting healing of such lesions (81). Hence, future murine and human studies to clarify the specific mechanisms underlying TGF-β fluctuation and functionality are needed. Furthermore, another important player in the matrix restructuring landscape are Matrix Metalloproteinases (MMPs), which directly control extracellular matrix degradation and collagen turnover. It’s also produced by some shared cell types which also produce TGF-β, such as macrophages and eosinophils. Its physiological mechanism operates as a paired equilibrium with tissue Inhibitors of metalloproteinases (TIMPS) (82). Hence a disruption in such balance resulting in altered MMP/TIMP ratio, contributes significantly towards tissue remodeling through fibrosis. During homeostasis, studies in fish model (Piaractus mesopotamicus) showed MMP2 and MMP9 activity is associated with rapid muscle growth in juvenile age, while MMP2 expression is significantly enhanced in muscle post cardiotoxin-mediated injury in murine model, demonstrating is regulatory and repair role in physiological states. In humans, it also regulates bone, blood vessel modeling. Similar to eosinophils, MMPs can be both pro and anti-inflammatory, facilitating both recruitment and clearance of immune cells through cleavage of inflammatory mediators (83). However, its subsets MMP9 are also present in Alzheimer’s, stroke and heart attacks in mice models alongside asthma and other inflammatory conditions.

### 4.2 Disease pathologies: uncontrolled neoplastic modeling in cancer

While eosinophils coordinate controlled tissue repair through IL-4, this same regenerative capacity can be hijacked in the tumor microenvironment (TME), leading to uncontrolled neoplastic modeling. Following research on the allergic responses of tumors over 70 years ago, studies observed a striking correlation between IgE mediated allergies and cancer, suggested by Ure in 1969 where the cancer group’s incidence of asthma was only 0.7% compared to the control group’s 2.5% (84, 85). Ure discovered an inverse correlation between the prevalence of IgE-mediated allergies and that of cancer in patients (84, 85), with a 15 fold (13 control patients with atopy against 1 cancer patient) decreased manifestation of IgE-mediated atopy in cancer patients (86). This observation supports a model whereby IgE may play a protective role against cancer development. Although the link between IgE and cancer incidence remains unclear, CD23b mediates IgE production and IgE antibody-dependent cellular phagocytosis; this axis was experimentally validated to actively drive tumor cell death via CD23b-IgE-mediated ADCP in human ovarian tumor cell cultures (87). This process is upregulated by IL-4, providing a hypothetical, yet unverified, mechanistic link that could explain this clinical correlation.

As stated in **Figure 2**, tumor sites contain a vast array of cells, within which eosinophils play important roles that are far more sophisticated and complex than previously thought. Tumor-infiltrating eosinophils have been observed and studied in many types of solid tumors (88), primarily lung, colorectal, breast, gastric, esophageal cancers and melanomas and this is very much an active area of research.

The recruitment of eosinophils from the blood to the tumor microenvironment (TME) is regulated through multiple pathways. The expression of CCL11, CCL24 and CCL5 by cells in the TME has the potential to activate CCR3 receptors on eosinophils and act as chemoattractant (89). Additionally, the migration of eosinophils has also been associated with the detection of damage-associated molecular patterns (DAMPs). These ‘danger signals’, which are expressed by dying cells and reflect low oxygen levels (hypoxia) at tumor sites, are caused through the expression of extracellular high mobility group box 1 (HMGB1) (90), which attracts eosinophils (91), as well as the expression of IL-33, a key inducer of Th2 responses (5, 90).

Similar to their role in the immune regulation of helminth infection, the function of eosinophils in cancer remains incompletely understood and highly context-dependent. Evidence supports both their pro- and anti-tumorigenic roles in the TME, influenced by tumor type, disease stage, and local immune signals, which are discussed below. Peripheral blood eosinophil counts are often assessed as a cancer prognosis indicator, but they may not accurately reflect intratumoral eosinophil function and are often altered by treatment effects and comorbidities. For instance, chemotherapy agents such as Intraperitoneal Paclitaxel (PTX), strikingly recruit eosinophils with levels going from 0.47% to 10%, in this case, it acts as a robust prognostic biomarker that is triggered by chemotherapy and correlates positively with overall survival from 17.3 months to 26.7 months (92). However, when patients are exposed to corticosteroids during cancer treatments, there is the risk of a masking effect. It induces rapid eosinopenia through apoptosis and chemotactic gradient inhibition, the decreased eosinophil count may not indicate sign of poor prognosis or aggressive disease. Additionally, where immune checkpoint inhibitors are administered, eosinophilia may present as an outcome of the treatment, rather than reflecting the patient’s intrinsic innate biological ‘anti-tumor’ state. It can also be confounded by allergies such as eczema, which leads to a baseline inflation, which complicates its interpretation as a prognostic marker, whether it’s a sign of anti-tumor response, the allergy itself, or a mix of both (93). Therefore, interpreting eosinophil count as a prognostic marker requires additional attention to adjust for potential confounders.

#### 4.2.1 Anti-tumorigenic and pro-inflammatory roles

**Figure 3:**
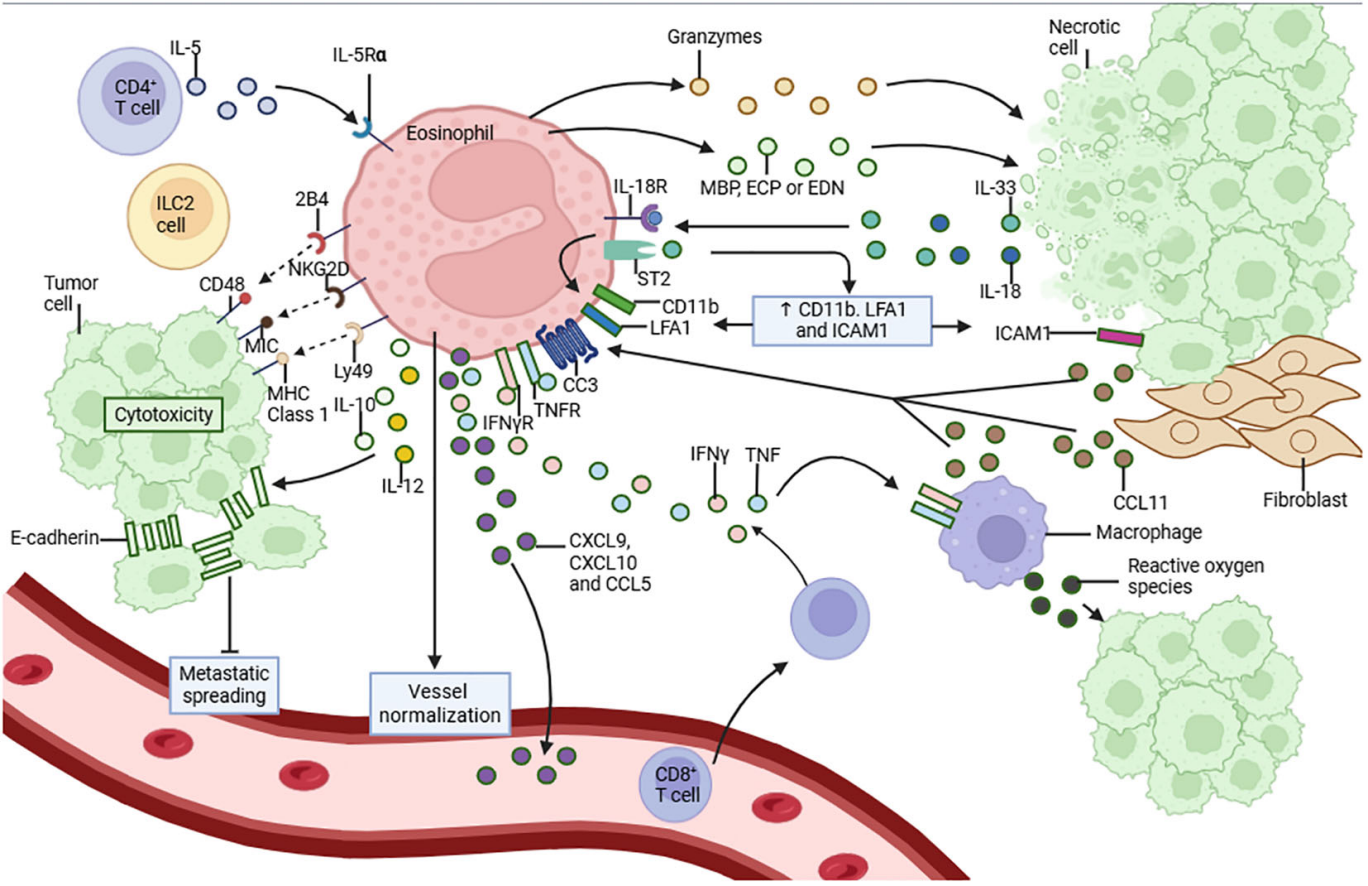
Schematic representation of anti-tumorigenic functions of eosinophils in the tumor microenvironment (TME), adapted from Grisaru-Tal et al. (2020). Eosinophils in the TME exhibit enhanced survival via IL-5/IL-5Rα signaling and recognize tumor cells through IL-33/ST2 and IL-18/IL-18R binding, accompanied by upregulation of adhesion factors CD11b, LFA1 and ICAM-1. They secrete cytotoxic granule proteins such as granzymes, MBP-1 and ECP/EDN proteins in response to IL-5, CCL11, IFN-γ, TNF and IL-33. Eosinophil-derived IL-10 and IL-12 enhance E-cadherin expression, IFN-γ promotes CD8^+^ T cell recruitment and cytotoxicity, and IFN-γ/TNF-activated eosinophils polarize ROS-secreting macrophages, collectively contributing to tumor cell death (94).

Despite its roles in uncontrolled neoplastic remodeling, eosinophils also exhibit anti-tumorigenic roles through a range of direct and indirect immune-modulatory mechanisms (as shown in **Figure 3**), resulting in a decrease in tumor size and metastatic potential and an increase in cytotoxicity and necrosis of tumor cells. Both tumor-associated eosinophilia within the tumor microenvironment (TME) and elevated peripheral blood eosinophil counts have been linked with improved clinical outcomes in specific cancer types, in particular colorectal carcinomas (CRC) (85), as well as melanomas, prostate, breast, and gastric cancers.

Once eosinophils have infiltrated the TME, the production of IL-5 - expressed largely by CD4^+^ T cells and type 2 innate lymphoid cells (ILC2s) is increased (94). Survival and activation of eosinophils is augmented through the binding of IL-5 to its receptor IL-5R on the eosinophil surface (95). Ikutani et al. showed that IL-5 deficient or IL-5 signaling-deficient mice, treated with anti-IL-5 monoclonal antibodies, have an increase in the growth and metastasis of tumors, and this was directly correlated with a decrease in the number of eosinophil cells infiltrating the TME, in a B16F10 melanoma model (95). However, whereas these results highlight the importance of IL-5 on eosinophil survival in the TME, other studies, for instance by Reichman et al. present results showing that eosinophil survival in the TME of colorectal cancer (CRC) is IL-5-independent (96). Further research on the mechanisms that promote eosinophil survival in the TME is necessary, particularly since these findings have potential applications to clinical cancer therapy.

Eosinophils have the ability to recognize tumor cells in the TME (94), through the binding of alarmins IL-33 and IL-18, expressed by tumor and necrotic cells, to their respective eosinophil surface receptors ST2 and IL-18R, respectively. Data from Lucarini et al. demonstrates that the IL-33/ST2 axis operates in a two-fold manner: it actively recruites intratumoral CD8^+^ T cells and activates NK cells (97), and enhances the expression of lymphocyte function-associated antigen 1 (LFA1) and CD11b integrins on eosinophil cell surface (98) and their binding to intracellular adhesion molecule 1 (ICAM-1) on tumor cells (94, 98). The CD11b/ICAM-1 interaction promotes cell-to-cell adhesion, which is typically followed by polarization and cytotoxic degranulation of MBP, eosinophil peroxidase, and granzymes inducing tumor cell death (94, 98). Degranulation is also triggered in response to IL-5, IFN-γ, TNF and CC-chemokine ligand 11 (CCL11), the latter also acting to limit angiogenesis of the tumor (99). In 2015, Gatault et al. observed the role of IL-18 in eosinophil-mediated death of Colo-205 cancerous cells of CRC through the upregulation of CD11a and ICAM-1 to promote cell contact and synaptic cytotoxicity (100), supporting the hypothesis of the anti-tumorigenic role of eosinophils in CRC.

Furthermore, adhesion of activated eosinophils to tumor cells prevents metastatic spread through the expression of IL-10 and IL-12 cytokines, as seen in prostate cancer (101), as well as through the IL-33/ST2 axis, as demonstrated by Lucarini et al. (97). This induces the potential upregulation of adhesion molecules such as E-cadherin, intercellular adhesion molecule-1 and vascular cell-adhesion protein-1 on eosinophil surfaces, inhibiting tumor cells from entering the blood flow and migrating to secondary sites (94, 101).

The secretion of TNF and IFN-γ in particular also plays a central role in the direct and indirect anti-tumorigenic functions of eosinophils (94). Reichman et al. used bulk RNA sequencing on intra-tumoral eosinophils in CRC to demonstrate that their anti-tumorigenic activities are associated with an IFN-γ-driven gene signature (96). This study further indicates that eosinophils differentiated with recombinant IL-18, which incurs the secretion of IFN-γ, exhibit a distinct gene expression profile associated with inflammation (96). In effect, this causes IFN-γ-activated eosinophils to resemble the phenotype of M1 macrophages and secrete ROS, nitric oxide and mitochondrial DNA, resulting in CD8+ T cell-independent tumor cell death. 

#### 4.2.2 Pro-tumorigenic roles

Pro-tumorigenic roles of eosinophils in cancer have also been studied, primarily in Hodgkin’s lymphoma, lung, ovarian and cervical cancers (5), for which eosinophilia is generally associated with a poor cancer prognosis. Evidence suggests that eosinophils may indirectly support tumor growth survival through mechanisms such as increased angiogenesis, secretion of growth factors and control of immune cells present within the TME (94).

Eosinophils located in the TME secrete pro-tumorigenic factors, such as VEGF-A and fibroblast growth factor (FGF-2), which have been associated with increased TME angiogenesis (5). Varey et al. further showed that the VEGF-A gene contains both pro- and anti-tumorigenic sequences and that alternative splicing of the gene leads to the pro-angiogenic isoform VEGF-A, which becomes dominant over anti-tumorigenic isoform VEGF-A_165_b in specific malignancies (102). Parallel mechanistic evidence indicates that this pro-tumorigenic angiogenic activity is subsequently amplified by the localized release of eosinophil-derived MBP-1.

Eosinophils further act as pro-tumorigenic cells through their immune-suppressing production of indoleamine 2,3-dioxygenase (IDO). IDO catabolizing enzyme is responsible for the inhibition of T and NK cell functions, and in cancer, provides a mechanism of immune-evasion through immunotolerance of tumor cells (103).

In addition to promotion of new blood vessels, pro-tumorigenic eosinophil roles have been associated with increased prevalence of metastasis through mechanisms involving lymphangiogenesis (97). While the formation of lymphatic vessels is primarily driven by VEGF-C and VEGF-D factors (5), the production of these factors by eosinophils has not yet been evaluated. However, recent advances by Li et al. have studied the role of eosinophil-secreted CCL6 in tumor metastasis (104). CCL6 binding to its receptor CCR1 induces the recruitment of tumor cells, promoting the migration of tumor cells and formation of metastatic niches (104). Their analysis established that inhibition of CCL6/CCR1 binding, reduces tumor-cells migration (104). Although this study adopted mouse models, and particularly applied to the study of bone cancer, inhibition of CCR1 and binding of CCR1 and CCL23 (the human homolog of CCL6 in mice) warrants further research. In a study of cervical cancer, Zhang et al. show that along with increased tumor-associated angiogenesis in cervical cancer, tumor-derived thymic stromal lymphopoietin promotes an increase in proliferation and migration of cervical cancer cells through the overexpression of IL-4, 5, 10 and 13 from activated eosinophils (105), while studies of lung cancer by Zaynagetdinoy et al. suggest that IL-5 is largely responsible for tumor metastasis (106). Although the downstream targets of eosinophils that are responsible for metastasis appear to be largely cancer-specific and are still being elucidated, eosinophils and their mediators have been, in general, associated with metastasis. It would therefore be interesting to explore eosinophil-depleting therapies in cancers in which eosinophil roles are pro-tumorigenic as a preventative measure against metastasis formation.

Although the roles of eosinophils in cancer has not been fully elucidated, recent research clearly highlights their roles in cancer as more than passive bystanders. There is evidence for both pro- and anti-tumorigenic roles, with mounting evidence suggesting that these sided roles are cancer type- and stage-specific. Reichman et al. suggest that the predisposition of eosinophils to act as either pro- or anti-tumor regulators is shaped by the TME (96). In their view, a TME characterized by strong innate stimulus and exposure to IFN-γ skews the balance of pro- and anti-tumor roles towards the anti-tumoral effects. This observation highlights the potent, context-dependent roles that eosinophils play in the TME and suggests their potential use as therapeutic targets for emerging cancer therapies.

### 4.3 Disease pathologies: eosinophilic gastrointestinal disorders (EGID)

EGIDs are a group of chronic and diverse intestinal diseases characterized by the infiltration and accumulation of eosinophils in the mucosa of any part of the gastrointestinal tract (107). EGIDs comprise eosinophilic esophagitis (EoE), as well as eosinophilic gastritis, colitis and gastroenteritis, which are non-esophageal EGIDs and are less prominent and less well understood. The review will focus on EoE as the primary disease leading to food bolus obstruction (108) and with its prevalence rapidly increasing worldwide over the last two decades (108). Although EoE is Th2-mediated and allergen-driven, the understanding of this inflammatory disorder remains limited, contrary to historical assumptions, clinical trials evaluating traditional allergy testing consistently failed to predict dietary triggers in adultEoE patients, demonstrating that these disorders operate via a non-IgE-mediated pathology (109). 

**Figure 4:**
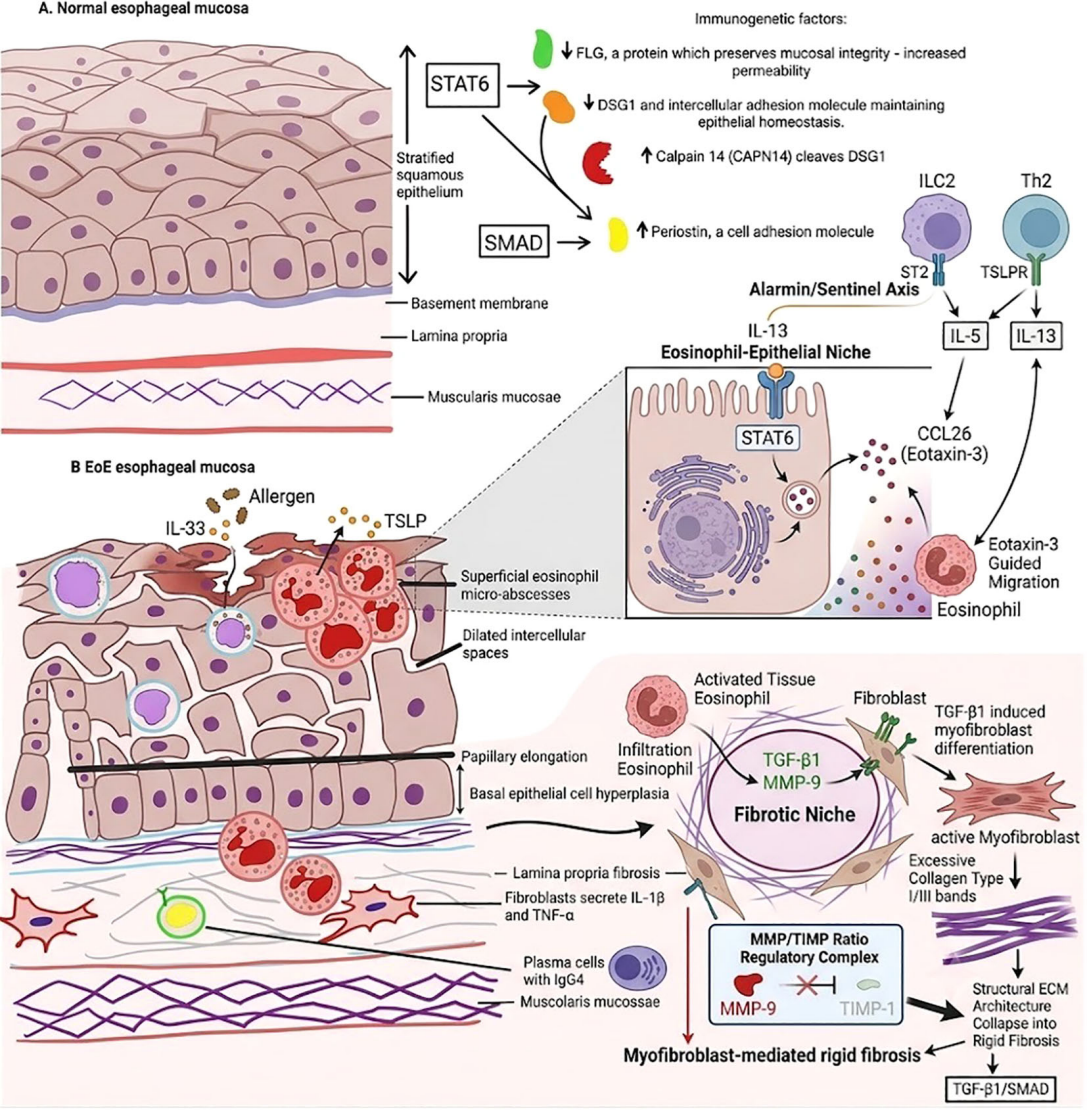
Spatial signaling networks and matrix-remodeling loops in Eosinophilic Esophagitis (EoE). **(A)** Normal esophageal mucosa compared with **(B)** pathological alterations defining EoE. Mucosal damage triggers an Alarmin/Sentinel Axis (IL-33/TSLP) that activates local ILC2 and Th2 cells to release IL-5 and IL-13. Within the Eosinophil-Epithelial Niche, IL-13 prompts epithelial STAT6 activation and the formation of a guided CCL26 (Eotaxin-3) spatial migration gradient. Infiltrating eosinophils establish a Fibrotic Niche by secreting TGF-β1 and MMP-9. TGF-β1 induces fibroblast differentiation into active myofibroblasts that deposit excessive Collagen Type I/III bands. This pathway is compounded by an uncoupled MMP/TIMP Ratio Regulatory Complex where unchecked MMP-9 activity drives structural ECM architectural collapse, culminating in myofibroblast-mediated rigid tissue fibrosis via downstream TGF-β1/SMAD signaling (93).

Under healthy homeostatic conditions, the esophagus is free of eosinophils, however, in EoE, eosinophils infiltrate the tissue, through IL-13-driven recruitment, and form superficial micro-abscesses. Alongside the elongation of papillary cells and dilation of intercellular spaces, these factors cause interference with the esophageal barrier, rendering patients more susceptible to pathogens and allergens (93). The presence of allergens initiate the Th2 inflammation cascade, leading to eosinophil recruitment, mediated by CCL26 (as opposed to CCL11 and CCL24 in asthma), and prolongs eosinophilic survival primarily in the basal layer of the epithelium and lamina propria, leading to expansion into the submucosa (110). Additionally, the eosinophilic-derived cationic proteins TGF-β1 and VCAM-1, cause tissue damage of the esophagus and fibrosis (111), as well as basal cell hyperplasia through action of TGF-β1 (111). IL-4, IL-5 and IL-13 cytokines, released by Th2 lymphocytes and induced eosinophils, are responsible for activation of the STAT-5 and STAT-6 pathways that modulate the expression of CCL26, periostin, desmoglein-1 (DSG-1) and calpain-14 (CAPN-14), which further promote eosinophilic infiltration, inflammation, epithelial damage and remodeling of the esophagus (110). The etiology of EoE remains unclear. Indeed, unlike allergic reactions in which the Th2 pathway promotes secretion of IgE immunoglobulin from B cells, there is no evidence of IgE involvement in EoE. The pathology of EoE has been schematically shown by Lim et al. in **Figure 4**.

Current EoE treatments include elimination diets aimed at removing the inflammation triggers, and pharmacological therapies, primarily proton pump inhibitors (PPIs) and swallowed topical corticosteroids (STCs), which target and downregulate gene expression of key EoE divers: CCL26 and IL-13 (93).

### 4.5 Physiological conditions: reproductive homeostasis and embryological modeling

If the eosinophil is the primary architect of tissue repair and the primary effector of tissue destruction, its role in reproductive homeostasis represents the synthesis of these functions. In the uterus, eosinophils must maintain homeostatic epithelial integrity while preparing for the ‘controlled inflammation’ of blastocyst implantation. This indicates that the eosinophil is not merely a cell of ‘health or disease,’ but is central to the original embryological modeling that precedes both. Although the roles of eosinophils are starting to be elucidated, eosinophil function in women’s health, and primarily in pregnancy are yet preliminary. Embryo implantation in early pregnancy is an immunological paradox (13), involving a blastocyst formed of maternal and paternal genes that becomes tolerated by the immune system and induces a cascade of events in the female body. Eosinophils are evidently found widespread in the female reproductive tract (112), notably in the uterus, and are involved in the development and homeostasis of uterine epithelial integrity. Based on tissue-resident profiling, eosinophils represent a highly abundant innate cell lineage in the mammalian uterus (3), Leading to the hypothesis that they prepare the local microenvironment for the controlled inflammation that accompanies blastocyst implantation. Characteristically, murine models demonstrate that the uterine endometrial eosinophil count fluctuates predictiably across the estrus cycle (13), with peaks of eosinophil numbers during estrus (ovulation) and metestrus (period marked by an increase in progesterone and decrease in estrogen), pointing to a regulatory role in cyclical tissue restructuring Suggestively, eosinophils are involved in regulation of the uterine cycle, and it is therefore possible to suggest a correlation between the number of eosinophils present in the uterus and the correct and timely occurrence of ovulation and changes to the endometrium, through a hormone balance, ready for blastocyst implantation. Kurganov (113) showed that the endometrial inability to accept implantation of a fertilized egg can be caused by the overly-dense eosinophilic infiltration of the uterine wall, with eosinophilic infiltration of the uterus promoted through estrogen-driven CCL5 expression, suggesting a potential positive feedback loop involving eosinophil is associated with infertility. Ono et al. identified distinct eosinophils clusters in 94% (17 of 18) of human ovarian endometrioma cases, compared to only 19% (3 of 16) in non-endometriotic ovarian tumors and no accumulation in preserved normal ovary structures. While these data strongly implicate eosinophils as histopathological drivers of endometriotic fibrosis, further functional studies are required to definitively establish whether this local remodeling directly contributes to endometriosis-associated clinical infertility (114, 115), the association of eosinophils with infertility is highly likely as it’s a major. This is further supported by Ono etal as the first to provide mechanistic insight into endometriotic lesions, where Granulocyte-macrophage colony-stimulating factor (GM-CSF) levels increased to 93.6 pg/ml compared to baseline, while Plasminogen activator inhibitor-1 expression increased 3 fold when co-cultured with eosinophil (116). However, such framework remains hypothetical and requires elucidation.

Additionally, although reasons remain unknown, some studies speculate eosinophils to play an important role in the progression of pregnancy, with measured urine concentrations of eosinophil derived neurotoxin (EDN) increasing until the second trimester (117). Pati et al. suggested a possible direct link between pregnancy and the presence of anti-tumor and pro-apoptotic factors (specifically EDN and human chronic gonadotropin (hCG)-associated factor) in pregnancy urine, however this correlation remains unclear and requires additional research in order to understand whether this is simply an indication of pregnancy-related inflammation as a result of embryo development, or rather, if EDN, and therefore eosinophils, hold a more novel and critical role in the immune regulation of pregnancy. It’s also important to note clinical cases of benralizumab application which depletes eosinophils, but did not affect pregnancy and did not show short-term effects (55, 118). However, these eosinophil depleted studies are case reports without mechanistic assessments, hence conclusions cannot be made until higher sample sizes are assessed with a dissective approach. Questions remain to assess whether there is truly an underlying mechanism for these findings whether the presence of eosinophils is essential for embryological development, or is it required in certain scenarios of disease-related pathogenesis.

Additionally, murine characterization indicates that eosinophils are critical coordinators of parturition; fduring mouse labor, they accumulate in the cervix andsecrete pro-inflammatory cytokines in response to systematic progesterone withdrawal. This localized influx works to disrupt the rigid collagen matrix, a requirement for ripening the delivery canal (119). Additionally, eosinophils are also implicated in the tissue and muscle remodeling post-partum, particularly following Caesarean-sections. However, the roles of eosinophils in uterine homeostasis and pregnancy are still unclear as eosinophil-deficient mice also exhibit normal fertility and parturition where IL-5 depletion to achieve eosinophil depletion resulted only in increased placenta size, and no change in fetal weight, and postnatal survival, most importantly did not affect viable pregnancies compared to control, remaining around 32% (120). A clinical case report by Manetz.S et al. indicate that a healthy pregnancy and complete eosinophil-depletion are not mutually exclusive, providing a proof-of-concept case where an eosinophil-deficient patient successfully carried and delivered a healthy infant. However, it should be reminded the high risk of confounding factors such as individual heterogeneity and sample size, as it may not be replicable. Therefore, conducting higher sample size and mechanistic studies are necessary to explore definitive role of eosinophils in fertility and pregnancy.

The journey of pregnancy, that involves at least two organisms’ immune systems is one of, if not, the most significant immunological tolerant challenges that the female body is capable of, and the roles of eosinophils in this process deserve further research. More than that, this review has shed light on the array of beneficiary and detrimental roles that eosinophils play in the immune system, which appear to be dependent on the composition of their microenvironment. It has been shown that through IL-4 mediation, eosinophils are vital innate cells in controlled tissue creation, remodeling and repair and, according to Kurane et al., even in organ formation (13). However, paradoxically to this constructive role, eosinophils are also fundamental effector cells in uncontrolled tissue remodeling and neoplastic development leading to cancer establishment. With the principles that destruction opposes foundation, and that eosinophils play central roles in both processes, it is possible that eosinophils are critical innate cells in the original modeling that occurs during pregnancy: embryo development. However, very little research has been conducted on the roles of eosinophils in Embryology, and current studies on embryological modeling is highly speculative and adversarial, requiring further research in embryological immunology.

## 5 Conclusion and future work

This review confirms that eosinophils occupy a central position in immune networks and, as such, being implicated in an increasingly diverse array of health and disease contexts. While further research on the roles of eosinophils in immune regulation is necessary and ongoing, it appears that IL-4, IL-5 and IFN-γ pathways may be especially important in eosinophil-mediated immune regulation of diseases, including — but not limited to — homeostasis, cancer, respiratory, reproductive, vascular, gastrointestinal, muscular and immune diseases.

Although their roles are crucial, it appears that the microenvironment of eosinophils is itself a key determinant of eosinophils’ immunological roles, whether pro- or anti-inflammatory, beneficial or detrimental, prognostically good or poor. Understanding the factors at work in the healthy eosinophilic microenvironment — and those that skew it in disease — is vital in order to devise effective treatments for eosinophil-associated diseases. It is due to their central role in immunity that treatment of eosinophil-based disorders must be targeted. For example, although IL-5 blocking monoclonal antibody is successfully used in the treatment of eosinophilic asthma, this strategy also works to greatly reduce airway tissue, periphery and bone-marrow eosinophils. This approach, through down-regulation of eosinophil numbers and functions, places the organism at loss of the numerously beneficial, pro-inflammatory and anti-tumorigenic roles that eosinophils also have to offer, in the regulation of diseases such as cancer, tissue repair and respiratory diseases, in which eosinophilia is associated with a good prognosis. Ideally, taking advantage of the pioneering of asthma IL-5 blocking antibody, future treatment strategies should be rather more targeted to diseases-specific mediators in order to prevent indiscriminate eosinophil ablation. This ambitious aim, while simply stated, may be accomplished through a better understanding of juxtaposed eosinophil roles in controlled tissue remodeling as well as tumorigenic — uncontrolled — modeling. This would also serve as for a stepping-stone to the study of the roles of eosinophils in embryology. Through understanding eosinophil functions in embryological tissue generation and formation, we can hope to understand their more global functions in development, homeostasis and disease states.

## References

[1] Lombardi C Berti A Cottini M . The emerging roles of eosinophils: Implications for the targeted treatment of eosinophilic-associated inflammatory conditions. Curr Res Immunol. (2022) 3:42–53. doi: 10.1016/j.crimmu.2022.03.002 35496822 PMC9040157

[2] Gelaw Y Asrie F Walle M Getaneh Z . The value of eosinophil count in the diagnosis of preeclampsia among pregnant women attending the University of Gondar Comprehensive Specialized Hospital. Northwest Ethiopia. 2021. (2022) 22:557. doi: 10.1186/s12884-022-04892-9 35820860 PMC9274180

[3] Weller PF Spencer LA . Functions of tissue-resident eosinophils. Nat Rev Immunol. (2017) 17:746–60. doi: 10.1038/nri.2017.95 28891557 PMC5783317

[4] Lee JJ Jacobsen EA Ochkur SI McGarry MP Condjella RM Doyle AD . Human versus mouse eosinophils: ‘that which we call an eosinophil, by any other name would stain as red’. J Allergy Clin Immunol. (2012) 130:572–84. doi: 10.1016/j.jaci.2012.07.025 22935586 PMC3496419

[5] Varricchi G Galdiero MR Loffredo S Lucarini V Marone G Mattei F . Eosinophils: The unsung heroes in cancer? Oncoimmunology. (2018) 7. doi: 10.1080/2162402x.2017.1393134 29308325 PMC5749653

[6] Kim HJ Jung Y . The emerging role of eosinophils as multifunctional leukocytes in health and disease. Immune Netw. (2020) 20. doi: 10.4110/in.2020.20.e24 32655972 PMC7327148

[7] Lombardi C Bagnasco D COVID-19 PG . Eosinophils, and biologicals for severe asthma. Front Allergy. (2022) 3:859376. doi: 10.3389/falgy.2022.859376 35769563 PMC9234863

[8] Rosenberg HF Dyer KD Foster PS . Eosinophils: changing perspectives in health and disease. Nat Rev Immunol. (2013) 13:9–22. doi: 10.1038/nri3341 23154224 PMC4357492

[9] Liu J Yang C Liu T Deng Z Fang W Zhang X . Eosinophils improve cardiac function after myocardial infarction. Nat Commun. (2020) 11. doi: 10.1038/s41467-020-19297-5 33328477 PMC7745020

[10] Travers J Rothenberg ME . Eosinophils in mucosal immune responses. Mucosal Immunol. (2015) 8:464–75. doi: 10.1038/mi.2015.2 25807184 PMC4476057

[11] Jung Y Rothenberg ME . Roles and regulation of gastrointestinal eosinophils in immunity and disease. J Immunol. (2014) 193:999–1005. doi: 10.4049/jimmunol.1400413 25049430 PMC4109658

[12] Hassani M Staveren S Grinsven E Bartels M Tesselaar K Leijte G . Characterization of the phenotype of human eosinophils and their progenitors in the bone marrow of healthy individuals. Haematologica. (2020) 105. doi: 10.3324/haematol.2019.219048 31101758 PMC7012463

[13] Kurane T Kawase F Morooka A Konno T . Spatio-temporal distribution of eosinophils in the mouse uterus during peri-implantation period. Okajimas Folia Anat Jpn. (2019) 96:49–56. doi: 10.2535/ofaj.96.49 31902831

[14] Wechsler ME Munitz A Ackerman SJ Drake MG Jackson DJ Wardlaw AJ . Eosinophils in health and disease: A state-of-the-art review. Mayo Clin Proc. (2021) 96:2694–707. doi: 10.1016/j.mayocp.2021.04.025 34538424

[15] Klion AD Ackerman SJ Bochner BS . Contributions of eosinophils to human health and disease. Annu Rev Pathol. (2020) 15:179–209. doi: 10.1146/annurev-pathmechdis-012419-032756 31977298 PMC7604902

[16] Spencer LA Szela CT Perez SA Kirchhoffer CL Neves JS Radke AL . Human eosinophils constitutively express multiple Th1, Th2, and immunoregulatory cytokines that are secreted rapidly and differentially. J Leukoc Biol. (2009) 85:117–23. doi: 10.1189/jlb.0108058 18840671 PMC2626765

[17] Acharya KR Ackerman SJ . Eosinophil granule proteins: form and function. J Biol Chem. (2014) 289:17406–15. doi: 10.1074/jbc.r113.546218 24802755 PMC4067173

[18] Doyle AD Jacobsen EA Ochkur SI McGarry MP Shim KG Nguyen DT . Expression of the secondary granule proteins major basic protein 1 (MBP-1) and eosinophil peroxidase (EPX) is required for eosinophilopoiesis in mice. Blood. (2013) 122:781–90. doi: 10.1182/blood-2013-01-473405 23736699 PMC3731932

[19] Huang L Appleton JA . Eosinophils in helminth infection: Defenders and dupes. Trends Parasitol. (2016) 32:798–807. doi: 10.1016/j.pt.2016.05.004 27262918 PMC5048491

[20] Jacobsen EA Helmers RA Lee JJ Lee NA . The expanding role(s) of eosinophils in health and disease. Blood. (2012) 120:3882–90. doi: 10.1182/blood-2012-06-330845 22936660 PMC3496950

[21] Ondari E Calvino-Sanles E First NJ Gestal MC . Eosinophils and bacteria, the beginning of a story. Int J Mol Sci. (2021) 22. doi: 10.3390/ijms22158004 34360770 PMC8347986

[22] GBDCRD C . Global, regional, and national deaths, prevalence, disability-adjusted life years, and years lived with disability for chronic obstructive pulmonary disease and asthma, 1990-2015: a systematic analysis for the Global Burden of Disease Study 2015. Lancet Respir Med. (2017) 5:691–706. 28822787 10.1016/S2213-2600(17)30293-X PMC5573769

[23] Woodruff PG Modrek B Choy DF Jia G Abbas AR Ellwanger A . T-helper type 2-driven inflammation defines major subphenotypes of asthma. Am J Respir Crit Care Med. (2009) 180:388–95. doi: 10.1164/rccm.200903-0392oc 19483109 PMC2742757

[24] Halwani R Al-Muhsen S Al-Jahdali H Hamid Q . Role of transforming growth factor-beta in airway remodeling in asthma. Am J Respir Cell Mol Biol. (2011) 44:127–33. doi: 10.1016/j.coph.2010.06.004 20525803

[25] Jacobsen EA Jackson DJ Heffler E Mathur SK Bredenoord AJ Pavord ID . Eosinophil knockout humans: Uncovering the role of eosinophils through eosinophil-directed biological therapies. Annu Rev Immunol. (2021) 39:719–57. doi: 10.1146/annurev-immunol-093019-125918 33646859 PMC8317994

[26] Jacobsen EA Doyle AD Colbert DC Zellner KR Protheroe CA LeSuer WE . Differential activation of airway eosinophils induces IL-13-mediated allergic Th2 pulmonary responses in mice. Allergy. (2015) 70:1148–59. 10.1111/all.12655 PMC4552595 26009788

[27] Jacobsen EA Zellner KR Colbert D Lee NA Lee JJ . Eosinophils regulate dendritic cells and Th2 pulmonary immune responses following allergen provocation. J Immunol. (2011) 187:6059–68. doi: 10.4049/jimmunol.1102299 22048766 PMC3375323

[28] MacKenzie M J D LA F P.S . Eosinophils promote allergic disease of the lung by regulating CD4(+) Th2 lymphocyte function. J Immunol. (2001) 167:3146–55. doi: 10.4049/jimmunol.167.6.3146 11544300

[29] Hammad H Lambrecht BN . The basic immunology of asthma. Cell. (2021) 184:2521–2. doi: 10.1016/j.cell.2021.02.016 33930297

[30] Padigel UM Lee JJ Nolan TJ SChad GA Abraham D . Eosinophils can function as antigen-presenting cells to induce primary and secondary immune responses to Strongyloides stercoralis. Infect Immun. (2006) 74:3232–38. doi: 10.1128/iai.02067-05 16714550 PMC1479274

[31] Spencer LA Weller PF . Eosinophils and Th2 immunity: contemporary insights. Immunol Cell Biol. (2010) 88:250–6. doi: 10.1038/icb.2009.115 20065995 PMC3589820

[32] Lucey DR Nicholson-Weller A Weller PF . Mature human eosinophils have the capacity to express HLA-DR. Proc Natl Acad Sci USA. (1989) 86:1348–51. doi: 10.1073/pnas.86.4.1348 2919183 PMC286687

[33] Flood-Page P Menzies-Gow A Phipps S Ying S Wangoo A Ludwig MS . Anti-IL-5 treatment reduces deposition of ECM proteins in the bronchial subepithelial basement membrane of mild atopic asthmatics. J Clin Invest. (2003) 112:1029–36. doi: 10.1172/jci17974 14523040 PMC198522

[34] Evans CM Fryer AD Jacoby DB Gleich GJ Costello RW . Pretreatment with antibody to eosinophil major basic protein prevents hyperresponsiveness by protecting neuronal M2 muscarinic receptors in antigen-challenged Guinea pigs. J Clin Invest. (1997) 100:2254–62. doi: 10.1172/jci119763 9410903 PMC508421

[35] Ochkur SI Jacobsen EA Protheroe CA Biechele TL Pero RS McGarry MP . Coexpression of IL-5 and eotaxin-2 in mice creates an eosinophil-dependent model of respiratory inflammation with characteristics of severe asthma. J Immunol. (2007) 178:7879–89. doi: 10.4049/jimmunol.178.12.7879 17548626

[36] Gleich GJ Adolphson CR Leiferman KM . The biology of the eosinophilic leukocyte. Annu Rev Med. (1993) 44:85–101. doi: 10.1146/annurev.med.44.1.85 8476270

[37] Gundel RH Letts LG Gleich GJ . Human eosinophil major basic protein induces airway constriction and airway hyperresponsiveness in primates. J Clin Invest. (1991) 87:1470–3. doi: 10.1172/jci115155 2010556 PMC295201

[38] G H HM B J J R L F B CJ D . Eosinophil diversity in asthma. Biochem Pharmacol. (2020) 179:113963. 32278006 10.1016/j.bcp.2020.113963

[39] Piliponsky AM Pickholtz D Gleich GJ Levi-Schaffer F . Human eosinophils induce histamine release from antigen-activated rat peritoneal mast cells. J Allergy Clin Immunol. (2001) 107:993–1000. doi: 10.1067/mai.2001.114656 11398076

[40] Ueki S Tokunaga T Melo RCN Saito H Honda K Fukuchi M . Charcot-Leyden crystal formation is closely associated with eosinophil extracellular trap cell death. Blood. (2018) 132:2183–7. doi: 10.1182/blood-2018-04-842260 30154112 PMC6238188

[41] Persson EK Verstraete K Heyndrickx I Gevaert E Aegerter H Percier JM . Protein crystallization promotes type 2 immunity and is reversible by antibody treatment. Science. (2019) 364. doi: 10.1126/science.aaw4295 31123109

[42] Minshall EM Leung DY Martin RJ Song YL Cameron L Ernst P . Eosinophil-associated TGF-beta1 mRNA expression and airways fibrosis in bronchial asthma. Am J Respir Cell Mol Biol. (1997) 17:326–33. doi: 10.1165/ajrcmb.17.3.2733 9308919

[43] Kanda A Yun Y Bui DV Nguyen LM Kobayashi Y Suzuki K . The multiple functions and subpopulations of eosinophils in tissues under steady-state and pathological conditions. Allergol Int. (2021) 70:9–18. doi: 10.1016/j.alit.2020.11.001 33243693

[44] Gleich GJ Loegering DA . Immunobiology of eosinophils. Annu Rev Immunol. (1984) 2:429–59. doi: 10.1146/annurev.immunol.2.1.429 6399849

[45] Price DB Rigazio A Campbell JD Bleecker ER Corrigan CJ Thomas M . Blood eosinophil count and prospective annual asthma disease burden: a UK cohort study. Lancet Respir Med. (2015) 3:849–58. doi: 10.1016/s2213-2600(15)00367-7 26493938

[46] Zhang J Qiu Y Liu Y . Eosinophil extracellular traps formation is correlated with cancer prognosis by tumor microenvironment remodeling. Sci Rep. (2025) 15:40209. doi: 10.1038/s41598-025-24010-x 41249786 PMC12623478

[47] Mesnil C Raulier S Paulissen G Xiao X Birrell MA Pirottin D . Lung-resident eosinophils represent a distinct regulatory eosinophil subset. J Clin Invest. (2016) 126:3279–95. doi: 10.1172/jci85664 27548519 PMC5004964

[48] Zhu C Weng Q Gao S Li F Li Z Wu Y . TGF-β signaling promotes eosinophil activation in inflammatory responses. Cell Death Dis. (2024) 15. doi: 10.1038/s41419-024-07029-2 39214980 PMC11364686

[49] Camelo A Rosignoli G Ohne Y Stewart RA Overed-Sayer C Sleeman MA . IL-33, IL-25, and TSLP induce a distinct phenotypic and activation profile in human type 2 innate lymphoid cells. Blood Adv. (2017) 1:577–89. doi: 10.1182/bloodadvances.2016002352 29296700 PMC5728348

[50] McBrien CN Menzies-Gow A . The biology of eosinophils and their role in asthma. Front Med. (2017) 4:93. doi: 10.3389/fmed.2017.00093 28713812 PMC5491677

[51] Arnold IC Munitz A . Spatial adaptation of eosinophils and their emerging roles in homeostasis, infection and disease. Nat Rev Immunol. (2024) 24:858–77. doi: 10.1038/s41577-024-01048-y 38982311

[52] Ecrement A Spasovski V Rolin G Barnig C . Exploring the diversity and emerging powers of eosinophil subpopulations. Allergy. (2025) 80:3012–26. doi: 10.1111/all.16631 40568764

[53] Elhusseini Z Hersh CP Wang J Yun JH . Pilot application of spatial transcriptomics for profiling eosinophils in chronic obstructive pulmonary disease lung tissues. Am J Respir Crit Care Med. (2025) 211:A4621. doi: 10.1164/ajrccm.2025.211.abstracts.a4621

[54] Gurtner A Borrelli C Gonzalez-Perez I Bach K Acar IE Núñez NG . Active eosinophils regulate host defence and immune responses in colitis. Nature. (2023) 615:151–7. doi: 10.1038/s41586-022-05628-7 36509106 PMC9977678

[55] Jackson DJ Korn S Mathur SK Barker P Meka VG Martin UJ . Safety of eosinophil-depleting therapy for severe, eosinophilic asthma: Focus on benralizumab. Drug Saf. (2020) 43:409–25. doi: 10.1007/s40264-020-00926-3 32242310 PMC7165132

[56] Leckie MJ Brinke A Khan J Diamant Z O’Connor BJ Walls CM . Effects of an interleukin-5 blocking monoclonal antibody on eosinophils, airway hyper-responsiveness, and the late asthmatic response. Lancet. (2000) 356:2144–8. doi: 10.1016/s0140-6736(00)03496-6 11191542

[57] Taliaferro W Sarles MP . The cellular reactions in the skin, lungs and intestine of normal and immune rats after injection with Nippostrongylus muris. J Infect Dis. (1939) 64:157–92. doi: 10.1093/infdis/64.2.157

[58] Tischendorf FW Brattig NW Buttner DW Pieper A Lintzel M . Serum levels of eosinophil cationic protein, eosinophil-derived neurotoxin and myeloperoxidase in infections with filariae and schistosomes. Acta Trop. (1996) 62:171–82. doi: 10.1016/s0001-706x(96)00038-1 9025985

[59] Singh A Batra JK . Role of unique basic residues in cytotoxic, antibacterial and antiparasitic activities of human eosinophil cationic protein. Biol Chem. (2011) 392:337–46. doi: 10.1515/bc.2011.037 21303303

[60] O’Connell AE Hess JA Santiago GA Nolan TJ Lok JB Lee JJ . Major basic protein from eosinophils and myeloperoxidase from neutrophils are required for protective immunity to Strongyloides stercoralis in mice. Infect Immun. (2011) 79:2770–8. 10.1128/IAI.00931-10 PMC3191984 21482685

[61] McCormick ML Metwali A Railsback MA Weinstock JV Britigan BE . Eosinophils from schistosome-induced hepatic granulomas produce superoxide and hydroxyl radical. J Immunol. (1996) 157:5009–15. doi: 10.4049/jimmunol.157.11.5009 8943408

[62] Venturiello SM Giambartolomei GH Costantino SN . Immune cytotoxic activity of human eosinophils against Trichinella spiralis newborn larvae. Parasite Immunol. (1995) 17:555–9. 10.1111/j.1365-3024.1995.tb00998.x 8817601

[63] Butterworth AE Remold HG Houba V David F D D P.H. . Antibody-dependent eosinophil-mediated damage to 51Cr-labeled schistosomula of Schistosoma mansoni. J Immunol. (1977) 118:2230–6. doi: 10.1084/jem.145.1.136 405426

[64] Knott ML Matthaei KI Giacomin PR Wang H Foster PS Dent LA . Impaired resistance in early secondary Nippostrongylus brasiliensis infections in mice with defective eosinophilopoeisis. Int J Parasitol. (2007) 37:1367–78. doi: 10.1016/j.ijpara.2007.04.006 17555758

[65] Ehrens A Hoerauf A Hübner MP . Eosinophils in filarial infections: Inducers of protection or pathology? Front Immunol. (2022) 13:983812. doi: 10.3389/fimmu.2022.983812 36389745 PMC9659639

[66] Bonne-Année S Nutman TB . Human innate lymphoid cells (ILCs) in filarial infections. Parasite Immunol. (2018) 40:10.1111/pim.12442. doi: 10.1111/pim.12442 28504838 PMC5685925

[67] Boyd A Ribeiro JMC Nutman TB . Human CD117 (cKit)+ innate lymphoid cells have a discrete transcriptional profile at homeostasis and are expanded during filarial infection. PloS One. (2014) 9:e108649. doi: 10.1371/journal.pone.0108649 25255226 PMC4177898

[68] Hall LR Mehlotra RK Higgins AW Haxhiu MA Pearlman E . An essential role for interleukin-5 and eosinophils in helminth-induced airway hyperresponsiveness. Infect Immun. (1998) 66:4425–30. doi: 10.1128/iai.66.9.4425-4430.1998 9712797 PMC108535

[69] Babu S Nutman TB . Immunology of lymphatic filariasis. Parasite Immunol. (2014) 36:338–46. doi: 10.1111/pim.12081 24134686 PMC3990654

[70] Rothenberg ME Hogan SP . The eosinophil. Annu Rev Immunol. (2006) 24:147–74. doi: 10.1146/annurev.immunol.24.021605.090720 16551246

[71] Ong RK Doyle RL . Tropical pulmonary eosinophilia. Chest. (1998) 113:1673–9. doi: 10.1378/chest.113.6.1673 9631810

[72] Tsanglao WR Nandan D Chandelia S Arya NK Sharma A . Filarial tropical pulmonary eosinophilia: a condition masquerading asthma, a series of 12 cases. J Asthma. (2019) 56:791–8. doi: 10.1080/02770903.2018.1490748 29969926

[73] Legrand F Herrick J Makiya M Ramanathan R Thompson R Rampertaap S . A randomized, placebo-controlled, double-blind pilot study of single-dose humanized anti-IL5 antibody (reslizumab) for the reduction of eosinophilia following diethylcarbamazine treatment of Loa loa infection. Clin Infect Dis. (2020) 73:e1624–e1631. doi: 10.1093/cid/ciaa1365 32910141 PMC8677597

[74] Herrick JA Metenou S Makiya MA Taylar-Williams CA Law MA Klion AD . Eosinophil-associated processes underlie differences in clinical presentation of loiasis between temporary residents and those indigenous to Loa-endemic areas. Clin Infect Dis. (2015) 60:55–63. doi: 10.1093/cid/ciu723 25234520 PMC4296126

[75] Fabre V Beiting DP Bliss SK Gebreselassie NG Gagliardo LF Lee NA . Eosinophil deficiency compromises parasite survival in chronic nematode infection. J Immunol. (2009) 182:1577–83. doi: 10.4049/jimmunol.182.3.1577 19155506 PMC3923382

[76] Gebreselassie NG Moorhead AR Fabre V Gagliardo LF Lee NA Lee JJ . Eosinophils preserve parasitic nematode larvae by regulating local immunity. J Immunol. (2012) 188:417–25. doi: 10.4049/jimmunol.1101980 22131328 PMC3244516

[77] Rotman HL Yutanawiboonchai W Brigandi RA Leon O Gleich GJ Nolan TJ . Strongyloides stercoralis: eosinophil-dependent immune-mediated killing of third stage larvae in BALB/cByJ mice. Exp Parasitol. (1996) 82:267–78. 10.1006/expr.1996.0034 8631378

[78] Goh YP Henderson NC Heredia JE Red Eagle A Odegaard JI Lehwald N . Eosinophils secrete IL-4 to facilitate liver regeneration. Proc Natl Acad Sci USA. (2013) 110:9914–9. doi: 10.1073/pnas.1304046110 23716700 PMC3683773

[79] Aoki A Hirahara K Kiuchi M Nakayama T . Eosinophils: Cells known for over 140 years with broad and new functions. Allergol Int. (2021) 70:3–8. doi: 10.1016/j.alit.2020.09.002 33032901

[80] Sumiyoshi K Nakao A Ushio H Mitsuishi K Okumura K Tsuboi R . Transforming growth factor-bβ1 suppresses atopic dermatitis-like skin lesions in NC/Nga mice. Clin Exp Allergy. (2002) 32:309–14. doi: 10.1046/j.1365-2222.2002.01221.x 11929498

[81] Deng Z Fan T Xiao C Tian H Zheng Y Li C . TGF-β signaling in health, disease and therapeutics. Sig Transduct Target Ther. (2024) 9:61. doi: 10.1038/s41392-024-01764-w 38514615 PMC10958066

[82] Arpino V Brock M Gill SE . The role of TIMPs in regulation of extracellular matrix proteolysis. Matrix Biol. (2015), 247–54. doi: 10.1016/j.matbio.2015.03.005 25805621

[83] Haro H Crawford HC Fingleton B MacDougall JR Shinomiya K Spengler DM . Matrix metalloproteinase-3–dependent generation of a macrophage chemoattractant in a model of herniated disc resorption. J Clin Invest. (2000) 105:133–41. doi: 10.1172/JCI7090 10642591 PMC377425

[84] Ure DM . Negative association between allergy and cancer. Scott Med J. (1969) 14:51–4. doi: 10.1177/003693306901400203 5812963

[85] Jensen-Jarolim E Achatz G Turner MC Karagiannis S Legrand F Capron M . AllergoOncology: the role of IgE-mediated allergy in cancer. Allergy. (2008) 63:1255–66. doi: 10.1111/j.1398-9995.2008.01768.x 18671772 PMC2999743

[86] Allegra J Lipton A Harvey H Luderer J Brenner D Mortel R . Decreased prevalence of immediate hypersensitivity (atopy) in a cancer population. Cancer Res. (1976) 36:3225–6. 975086

[87] Karagiannis SN Bracher MG Hunt J McCloskey N Beavil RL Beavil AJ . IgE-antibody-dependent immunotherapy of solid tumors. J Immunol. (2007) 179:2832–43. doi: 10.4049/jimmunol.179.5.2832 17709497

[88] Prizment AE Vierkant RA Smyrk TC Tillmans LS Lee JJ Sriramarao P . Tumor eosinophil infiltration and improved survival of colorectal cancer patients: Iowa Women’s Health Study. Mod Pathol. (2016) 29:516–27. doi: 10.1038/modpathol.2016.42 26916075 PMC4848192

[89] Lorena SC Oliveira DT Dorta RG Landman G Kowalski LP . Eotaxin expression in oral squamous cell carcinomas with and without tumour associated tissue eosinophilia. Oral Dis. (2003) 9:279–83. doi: 10.1034/j.1601-0825.2003.00958.x 14629326

[90] Bertheloot D Latz E . HMGB1, IL-1alpha, IL-33 and S100 proteins: dual-function alarmins. Cell Mol Immunol. (2017) 14:43–64. doi: 10.1038/cmi.2016.34 27569562 PMC5214941

[91] Lotfi R Lee JJ Lotze MT . Eosinophilic granulocytes and damage-associated molecular pattern molecules (DAMPs): role in the inflammatory response within tumors. J Immunother. (2007) 30:16–28. doi: 10.1097/01.cji.0000211324.53396.f6 17198080

[92] Matsumiya M Kurashina K Ohzawa H Takahashi R Suizu E Takahashi K . Role of intraperitoneal paclitaxel in eosinophil activation and recruitment in the peritoneal cavity and anti-tumor effects against peritoneal metastasis from gastric cancer. JCO. (2025) 43:471. doi: 10.1200/JCO.2025.43.4_suppl.471

[93] Lim AH Wong S Nguyen NQ . Eosinophilic esophagitis and IgG4: Is there a relationship? Dig Dis Sci. (2021) 66:4099–108. doi: 10.1007/s10620-020-06788-0 33534011

[94] Grisaru-Tal S Itan M Klion AD Munitz A . A new dawn for eosinophils in the tumour microenvironment. Nat Rev Cancer. (2020) 20:594–607. doi: 10.1038/s41568-020-0283-9 32678342

[95] Ikutani M Yanagibashi T Ogasawara M Tsuneyama K Yamamoto S Hattori Y . Identification of innate IL-5-producing cells and their role in lung eosinophil regulation and antitumor immunity. J Immunol. (2012) 188:703–13. doi: 10.4049/jimmunol.1101270 22174445

[96] Reichman H Itan M Rozenberg P Yarmolovski T Brazowski E Varol C . Activated eosinophils exert antitumorigenic activities in colorectal cancer. Cancer Immunol Res. (2019) 7:388–400. doi: 10.1158/2326-6066.cir-18-0494 30665890

[97] Lucarini V Ziccheddu G Macchia I Sorsa V Peschiaroli F Buccione C . IL-33 restricts tumor growth and inhibits pulmonary metastasis in melanoma-bearing mice through eosinophils. Oncoimmunology. (2017) 6. doi: 10.1080/2162402x.2017.1317420 28680750 PMC5486175

[98] Andreone S Spadaro F Buccione C Mancini J Tinari A Sestili P . IL-33 promotes CD11b/CD18-mediated adhesion of eosinophils to cancer cells and synapse-polarized degranulation leading to tumor cell killing. Cancers (Basel). (2019) 11. doi: 10.3390/cancers11111664 31717819 PMC6895824

[99] Xing Y Tian Y Kurosawa T Matsui S Touma M Yanai T . CCL11-induced eosinophils inhibit the formation of blood vessels and cause tumor necrosis. Genes Cells. (2016) 21:624–38. doi: 10.1111/gtc.12371 27169545

[100] Gatault S Delbeke M Driss V Sarazin A Dendooven A Kahn JE . IL-18 is involved in eosinophil-mediated tumoricidal activity against a colon carcinoma cell line by upregulating LFA-1 and ICAM-1. J Immunol. (2015) 195:2483–92. doi: 10.4049/jimmunol.1402914 26216891

[101] Furbert-Harris PM Parish-Gause D Hunter KA Vaughn TR Howland C Okomo-Awich J . Activated eosinophils upregulate the metastasis suppressor molecule E-cadherin on prostate tumor cells. Cell Mol Biol (Noisy-le-grand. (2003) 49:1009–16. 14682382

[102] Varey AH Rennel ES Qiu Y Bevan HS Perrin RM Raffy S . VEGF 165 b, an antiangiogenic VEGF-A isoform, binds and inhibits bevacizumab treatment in experimental colorectal carcinoma. Br J Cancer. (2008) 98:1366–79. doi: 10.1038/sj.bjc.6604308 18349829 PMC2361696

[103] Tanizaki Y Kobayashi A Toujima S Shiro M Mizoguchi M Mabuchi Y . Indoleamine 2,3-dioxygenase promotes peritoneal metastasis of ovarian cancer by inducing an immunosuppressive environment. Cancer Sci. (2014) 105:966–73. doi: 10.1111/cas.12445 24826982 PMC4317851

[104] Li F Du X Lan F Li N Zhang C Zhu C . Eosinophilic inflammation promotes CCL6-dependent metastatic tumor growth. Sci Adv. (2021) 7. doi: 10.1126/sciadv.abb5943 34039594 PMC8153717

[105] Zhang B Wei CY Chang KK Yu JJ Zhou WJ Yang HL . TSLP promotes angiogenesis of human umbilical vein endothelial cells by strengthening the crosstalk between cervical cancer cells and eosinophils. Oncol Lett. (2017) 14:7483–8. doi: 10.3892/ol.2017.7121 29344192 PMC5755249

[106] Zaynagetdinov R Sherrill TP Gleaves LA McLoed AG Saxon JA Habermann AC . Interleukin-5 facilitates lung metastasis by modulating the immune microenvironment. Cancer Res. (2015) 75:1624–34. doi: 10.1158/0008-5472.can-14-2379 25691457 PMC4401663

[107] Votto M Filippo M Olivero F Raffaele A Cereda E Amici M . Malnutrition in eosinophilic gastrointestinal disorders. Nutrients. (2020) 13. doi: 10.3390/nu13010128 33396413 PMC7824578

[108] Navarro P Arias A Arias-Gonzalez L Laserna-Mendieta EJ Ruiz-Ponce M Lucendo AJ . Systematic review with meta-analysis: the growing incidence and prevalence of eosinophilic oesophagitis. Aliment Pharmacol Ther. (2019) 49:1116–25. doi: 10.1111/apt.15231 30887555

[109] Philpott H Nandurkar S Royce SG Thien F Gibson PR . Allergy tests do not predict food triggers in adult patients with eosinophilic oesophagitis. Aliment Pharmacol Ther. (2016) 44:223–33. doi: 10.1111/apt.13676 27247257

[110] Clayton F Peterson K . Eosinophilic esophagitis: Pathophysiology and definition. Gastrointest Endosc Clin N Am. (2018) 28:1–14. 29129294 10.1016/j.giec.2017.07.011

[111] Straumann A Conus S Grzonka P Kita H Kephart G Bussmann C . Anti-interleukin-5 antibody treatment (mepolizumab) in active eosinophilic oesophagitis. Gut. (2010) 59:21–30. doi: 10.1136/gut.2009.178558 19828470

[112] Sferruzzi-Perri AN Robertson SA Dent LA . Interleukin-5 transgene expression and eosinophilia are associated with retarded mammary gland development in mice. Biol Reprod. (2003) 69:224–33. doi: 10.1095/biolreprod.102.010611 12620930

[113] Kurganov SA . Uterine eosinophils and infertility in the rat. Ross Fiziol Zh Im I M Sechenova. (2010) 96:138–46. 20432721

[114] Bonavina G Taylor HS . Endometriosis-associated infertility: From pathophysiology to tailored treatment. Front Endocrinol (Lausanne). (2022) 13:1020827. doi: 10.3389/fendo.2022.1020827 36387918 PMC9643365

[115] Vicetti Miguel RD Quispe Calla NE Dixon D Foster RA Gambotto A Pavelko SD . IL-4–secreting eosinophils promote endometrial stromal cell proliferation and prevent Chlamydia -induced upper genital tract damage. Proc Natl Acad Sci USA. (2017) 114. doi: 10.1073/pnas.1621253114 28765368 PMC5565408

[116] Ono Y Tanaka K Sato E Ito M Zhang D Honda M . Activated eosinophil plays a role in promoting fibrosis in endometriotic lesion. Sci Rep. (2025) 15:28015. doi: 10.1038/s41598-025-13855-x 40745369 PMC12313958

[117] Pati S Lee Y Samaniego F . Urinary proteins with pro-apoptotic and antitumor activity. Apoptosis. (2000) 5:21–8. doi: 10.1023/a:1009629424297 11227487

[118] Ozden G Pınar Deniz P . May mepolizumab used in asthma correct subfertility? Ann Med. (2021) 53:456–8. doi: 10.1080/07853890.2021.1900591 33739210 PMC7993371

[119] Timmons BC Fairhurst AM Mahendroo MS . Temporal changes in myeloid cells in the cervix during pregnancy and parturition. J Immunol. (2009) 182:2700–7. doi: 10.4049/jimmunol.0803138 19234164 PMC2752643

[120] Robertson SA Mau VJ Young IG Matthaei KI . Uterine eosinophils and reproductive performance in interleukin 5-deficient mice. J Reprod Fertil. (2000) 120:423–32. doi: 10.1530/jrf.0.1200423 11058459

